# Clinico-epidemiological insights and treatment practices of bovine lumpy skin disease in Bangladesh

**DOI:** 10.1371/journal.pone.0353682

**Published:** 2026-07-10

**Authors:** Md. Khalid Hasan Sumon, Sumya Binte Salam, Jesmin Sultana, Sharmin Aqter Rony, Farzana Yeasmin, AKM Anisur Rahman, Md. Amimul Ehsan, Md. Aminul Islam

**Affiliations:** 1 Department of Medicine, Immunogenomics and Alternative Medicine (IAM) Laboratory, Bangladesh Agricultural University, Mymensingh, Bangladesh; 2 Department of Parasitology, Faculty of Veterinary Sciences, Bangladesh Agricultural University, Mymensingh, Bangladesh; 3 Department of Medicine, Epidemiology and Preventive Medicine Laboratory, Bangladesh Agricultural University, Mymensingh, Bangladesh; Central Laboratory for Evaluation of Veterinary Biologics, Agricultural Research Center, EGYPT

## Abstract

**Background:**

Lumpy skin disease (LSD) is an emerging viral disease of cattle in Bangladesh, first reported in 2019. Given the critical role of veterinarians in disease detection, management, and control, it is essential to evaluate their knowledge, attitudes, and practices (KAP) regarding the clinical and epidemiological aspects of LSD and its treatment strategies.

**Methods:**

A cross-sectional survey was conducted among early-career veterinarians using a cluster random sampling technique to assess epidemiological insights and clinical practices regarding the LSD outbreak in the field.

**Results:**

All veterinary practitioners were aware of LSD, and 83.67% had previously managed LSD cases. Approximately 92% of cases of LSD are diagnosed clinically, primarily by observing circumscribed skin nodules, either alone or with other characteristic lesions. Nearly 69% considered clinical signs alone sufficient for diagnosis, although 73.62% reported diagnostic confusion with other diseases, including cowpox (29%) and papillomatosis (21%). Various treatment strategies were reported, with 43.22% of veterinarians supporting the use of antibiotics, and 60.5% using antibiotics in combination with other drugs. Notably, 62.06% recognized the potential for antibiotic resistance due to frequent use during LSD treatment. While 55.78% observed no drug-related side effects, 81.16% acknowledged occasional adverse effects. Regarding epidemiological perceptions, 85.56% believed that LSD affects cattle of all ages, 94.67% associated it with both sexes, and 67.03% reported no breed predisposition. Most (75.28%) linked disease occurrence to the hot and humid season. Perceived modes of transmission included mechanical transmission by arthropod vectors (33.69%), direct contact (22.19%), and a combination of both (16.84%).

**Conclusion:**

Bangladeshi veterinarians demonstrated sound knowledge, positive attitudes, and practical experience with LSD that aligns with current global scientific understanding. However, gaps remain in differential diagnosis and antimicrobial stewardship, warranting targeted training and policy interventions to address these needs.

## Introduction

Lumpy skin disease (LSD) is an emerging transboundary viral disease of cattle and buffaloes, caused by the LSD virus (LSDV), a member of the genus *Capripoxvirus* within the family *Poxviridae* [1,2]. Clinically, LSD is characterized by round, circumscribed skin nodules, fever, lymphadenopathy, limb and ventral body swelling, nasal discharge, and lacrimation. However, the severity and expression of clinical signs vary among affected animals [3]. The disease causes significant economic losses due to decreased milk production, reduced fertility, calf mortality, damage to hide quality, weight loss, and treatment costs [4,5]. Given its complex transmission dynamics and substantial economic impact, the World Organisation for Animal Health (WOAH, formerly OIE) classifies LSD as a notifiable transboundary animal disease [6].

LSDV is transmitted mainly through blood-feeding arthropod vectors (e.g., mosquitoes, biting flies, and ticks), but can also spread via direct contact, secretions, milk, or contaminated veterinary instruments [7]. The incidence of LSD tends to increase during the wet season and under communal grazing conditions, when vector abundance is high [8]. In Bangladesh, cattle constitute the largest livestock population (approximately 25.7 million) and are the species most susceptible to LSD, experiencing the greatest clinical and economic impacts [9,10].

First identified in Zambia in 1929, LSDV gradually spread throughout sub-Saharan Africa, the Middle East, Southeastern Europe, Central Asia, and, more recently, South Asia and China [11]. Since 2012, outbreaks have been frequently reported in the Middle East and West Asia [12]. The first outbreak in Europe occurred in Greece in 2015, from where it spread rapidly across Southeastern Europe [13]. Currently, LSD is endemic in many countries across Africa, the Middle East, parts of Central and South Asia, and Turkey [14].

In Bangladesh, LSD was first reported in mid-2019 among cattle in the Anwara, Karnaphuli, and Patiya upazilas of the Chattogram district [15]. The Department of Livestock Services (DLS) was notified of these suspected cases on July 21, 2019, and the diagnosis was confirmed on August 22, 2019, using RT-PCR [16]. Bangladesh officially notified the WOAH of the outbreak on September 15, 2019 [15]. By December 2019, the disease had spread nationwide [17]. As of January 14, 2020, over 568,470 cases were reported to DLS, though actual case numbers are likely higher due to underreporting and a shortage of trained veterinary personnel [18].

Early-career veterinarians are often the first responders in the field and are heavily involved in diagnosing and treating LSD. As such, their knowledge, attitudes, and practices (KAP) play a critical role in disease recognition, reporting, and control. The KAP framework assesses individual understanding, perceptions, and behaviors related to a specific issue and is widely used in public and animal health research [19]. Since LSD is a relatively new disease in Bangladesh, understanding veterinarians’ knowledge about its epidemiology, transmission, diagnosis, and treatment, as well as their attitudes and practical approaches, is crucial for effective disease control.

Despite growing evidence on the epidemiology and economic impact of lumpy skin disease (LSD), important gaps remain in understanding field-level management practices, particularly the knowledge, attitudes, and practices (KAP) of veterinarians in Bangladesh. Existing studies have largely focused on outbreak descriptions and economic assessments, with limited attention to how veterinary professionals diagnose, treat, and manage LSD under routine field conditions. Addressing this gap is essential for improving disease control strategies and rational antimicrobial use. Therefore, the present study aimed to assess the knowledge, attitudes, and practices of veterinarians regarding LSD in Bangladesh, and also to provide context-specific insights into field-level practices and variability in management approaches among veterinarians. This study offers novel evidence to inform targeted interventions and strengthen LSD control programs in Bangladesh.

## Materials and methods

### Ethics statement

The study protocol was reviewed and approved by the Animal Welfare and Experimentation Ethics Committee (approval No: AWEEC/BAU/2022(2)/42(b), dated July 27, 2022) of Bangladesh Agricultural University, Mymensingh, Bangladesh. Survey participants verbally agreed that the personal information we collected would be kept confidential. All potentially identifiable information and responses of the participants were anonymized before manuscript submission.

### Study design and study population

A cross-sectional questionnaire-based survey was conducted between 28/07/2022 and 30/06/2023. The study population comprised early-career veterinarians engaged in clinical practice across various Upazilas (sub-districts) in Bangladesh. As the Upazila is the smallest veterinary service providing unit in the country, it is typically the entry point for veterinary professionals through both government and non-governmental employment. Young veterinarians were selected as the target population because they are generally more actively involved in hands-on clinical work, unlike senior professionals who often undertake administrative responsibilities. This demographic was therefore considered most appropriate for assessing current clinical knowledge, attitudes, and practices regarding LSD.

### Sample size determination and sampling

The minimum required sample size for this cross-sectional study was calculated using the formula:

n = (Z^2^ × P(1 − P)/d^2^), where Z represents the standard normal deviate at a 95% confidence level (1.96), P is the expected proportion of the population with adequate knowledge (assumed at 0.5 for maximum sample size), and d is the desired precision (0.05). This resulted in a sample size of n = 384 [20]. To account for potential non-responses or incomplete questionnaires, an additional 4% (n = 14) was added, resulting in a final target of 398 participants.

**Fig 1:**
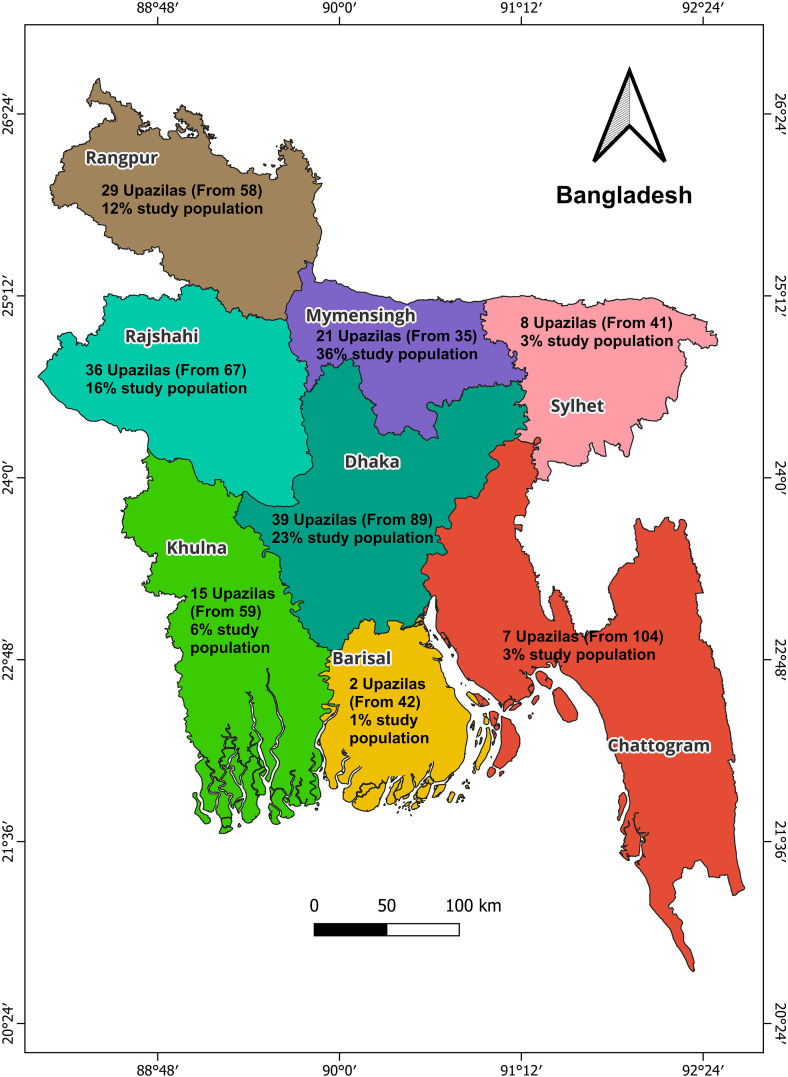
Map of Bangladesh showing the distribution of workplaces of the veterinarians surveyed (The map was generated using QGIS (version 3.36.1) with district boundary shapefiles obtained from the GADM database [22]).

A cluster random sampling technique was employed. In this approach, the primary sampling units were the veterinarians working in Upazilas, while the unit of analysis remained the individual veterinarian. A total of 157 Upazilas were randomly selected from a comprehensive list of 495 Upazilas across Bangladesh (Fig 1) using a random number generator in Microsoft Excel 2010 [21]. All veterinarians working in the government veterinary hospitals of the selected Upazilas were invited to participate in the survey. Face-to-face interviews were conducted with every invited individual who agreed to take part, which included government veterinary officers, intern veterinarians, and private veterinary practitioners stationed in those areas.

### Questionnaire design

A structured, closed-ended questionnaire was developed based on previous literature and the study objective to assess the knowledge, attitudes, and practices (KAP) of veterinarians regarding LSD. The questionnaire comprised three major sections: demographic information, knowledge and practices, and knowledge and attitudes.

The primary objectives of the KAP tool were to evaluate veterinarians’ understanding of LSD’s etiology, epidemiology, transmission dynamics, and diagnostic practices. Additional items focused on clinical expertise and field practices, including differential diagnoses, perceived disease consequences, case experience, and treatment protocols.

The questionnaire was reviewed by subject-matter experts for content validity and was pilot-tested on a small group of veterinarians outside the study population to ensure clarity and consistency. Since the questionnaire primarily consisted of independent knowledge, perception, and practice items rather than multi-item scales designed to measure a single latent construct, formal reliability coefficients were not calculated, but internal consistency and comprehension were carefully assessed during the pretesting phase. Based on expert feedback and pilot testing, the questionnaire was revised to enhance clarity and relevance.

The final version of the questionnaire consisted of three sections:

i Demographic Information: This section included variables such as gender, age, workplace, professional designation, and employment status.
ii Knowledge and Practice: This section included five questions addressing veterinarians’ familiarity with LSD clinical cases, recognition of clinical signs, differential diagnoses, treatment strategies, and case outcomes.
iii Knowledge and Attitude: This section contained nine items that assessed respondents’ opinions on the epidemiology and transmission of LSD, the importance of confirmatory diagnosis, and perceptions about antibiotic use and antimicrobial resistance.

### Data management and analysis

Data were reviewed for completeness and consistency before being entered into Microsoft Excel 2010. Demographic variables (e.g., age, sex, employment status, and years of service) were categorized accordingly. KAP-related variables were classified based on responses concerning clinical signs, differential diagnoses, treatment protocols, epidemiology, modes of transmission, and overall experience managing LSD cases. After data cleaning and validation (including addressing missing or inconsistent entries), descriptive statistics were performed using the R statistical software (v4.5.0). For each KAP category, correct or appropriate responses were aggregated. The percentage of correct responses was calculated by dividing the number of correct responses for a specific item by the total number of valid responses, and then multiplying by 100 to determine the mean percentage level across each KAP domain. These percentage scores provided an overview of the participants’ overall knowledge, attitudes, and practices related to LSD. To explore associations between demographic characteristics and key knowledge and attitude items, chi-square tests of independence were performed, with Fisher’s exact test applied where expected cell frequencies were less than five. Professional category (government veterinarian, private practitioner, or freshers) served as the primary grouping variable. For binary outcome variables, crude odds ratios (OR) with 95% confidence intervals (CI) were estimated using binary logistic regression. All comparative analyses are exploratory, given the demographic homogeneity of the sample. Statistical significance was set at p < 0.05. All analyses were conducted in R (v4.5.0).

## Results and discussion

### Characteristics of participant veterinarians

A total of 398 veterinarians participated in the study, of whom 283 (71.11%) were male, and 115 (28.89%) were female. Descriptive statistics of participant characteristics are presented in Table 1. The majority of respondents were young professionals aged between 21 and 29 years, with a substantial proportion clustered between the ages of 23 and 25. Specifically, participants aged 23, 24, and 25 years represented 21.11%, 35.68%, and 19.60% of the total sample, respectively.

**Table 1:** Socio-demographic characteristics and service modalities of veterinarians (N = 398).

Attribute	Category	Number	Percentage (%) [95% CIs]
**Sex**	Male	283	71.11 [66.47 to 75.34]
	Female	115	28.89 [24.66 to 33.53]
**Age (years)**	21-23 (1^st^ Quartile)	104	26.13 [22.06 to 30.66]
	24 (Median)	142	35.68 [31.13 to 40.50]
	25 (3^rd^ Quartile)	78	19.60 [16.00 to 23.78]
	26-29	74	18.59 [15.08 to 22.71]
**Employer/Job type**	Govt. vet	40	10.05 [7.47 to 13.40]
	Private vet	96	24.12 [20.00 to 28.63]
	Fresh graduates	262	65.82[61.04 to 70.32]
**Mode of Service**	Telemedicine	35	8.79 [6.39 to 11.98]
	On Farm	137	34.42 [29.92 to 39.22]
	On Clinic	124	31.16 [26.80 to 35.87]
	Both telemedicine and On Clinic	13	17.59 [14.16 to 21.63]
	Both telemedicine and On Farm	70	3.27 [1.92 to 5.51]
	Others	19	4.77 [3.08 to 7.34]

Regarding professional status, the majority of the respondents (65.82%) had recently completed their graduation, while 24.12% were employed as private practitioners, and only 10.55% responders were government-employed veterinarians. In terms of service delivery during the COVID-19 pandemic, 34.42% of veterinarians reported providing in-person field services, while 31.16% worked exclusively in clinics. Additionally, 17.59% of respondents delivered veterinary services both physically and remotely via mobile phone communication or telemedicine (Table 1).

The demographic profile of participants in this study highlights a predominance of young veterinary professionals, with the majority aged between 23 and 25 years. This finding aligns with the recent expansion of veterinary education in Bangladesh and similar low- and middle-income countries (LMICs), where increased graduation rates have contributed to a younger workforce entering the profession [23]. The high percentage of recent graduates (65.82%) further underscores the early-career composition of the sample. While such a demographic may bring enthusiasm and adaptability, it may also reflect limited field experience, potentially affecting their confidence and decision-making in complex clinical scenarios [24].

The professional distribution reveals that private practitioners constitute a significant proportion (24.12%) of the veterinary service workforce, reflecting the growing privatization of animal health services in the country. Conversely, the relatively low number of government veterinarians (10.55%) could be indicative of limited recruitment in the public sector operations in veterinary service delivery. Similar trends have been observed in other LMICs, where public veterinary services have become increasingly under-resourced, shifting the burden of care toward private and informal providers [14,25].

Service delivery modalities during the COVID-19 pandemic varied considerably among participants. Approximately one-third (34.42%) continued field-based services despite the heightened risk of exposure, indicating strong professional commitment and the essential nature of veterinary care even during public health emergencies. The notable use of telemedicine (17.59%), though still limited, suggests a nascent transition toward digital health solutions in veterinary practice. This echoes global trends in the adoption of telehealth, driven by pandemic-related restrictions and the need for continued service provision [26]. However, the modest uptake in this study points to possible challenges such as inadequate digital infrastructure, lack of regulatory frameworks, or client unfamiliarity with remote consultations [27].

Overall, these findings emphasize the importance of supporting early-career veterinarians through targeted training and mentorship, especially in field-based disease management and telehealth implementation. Strengthening the capacity of public veterinary services and integrating digital tools into routine care could enhance the resilience and reach of the veterinary sector, particularly in the face of future public health crises. Further studies are warranted to explore the effectiveness, feasibility, and acceptance of telemedicine in the veterinary context in Bangladesh and similar settings.

### Knowledge of veterinarians on the LSD outbreak

Though it is a recently emerged disease, all 398 participating veterinarians reported being aware of LSD, indicating 100% baseline familiarity with the disease. A majority (83.67%; n = 333) had directly managed LSD cases in the field, while 16.33% (n = 65) had no prior experience with LSD outbreak management.

**Fig 2:**
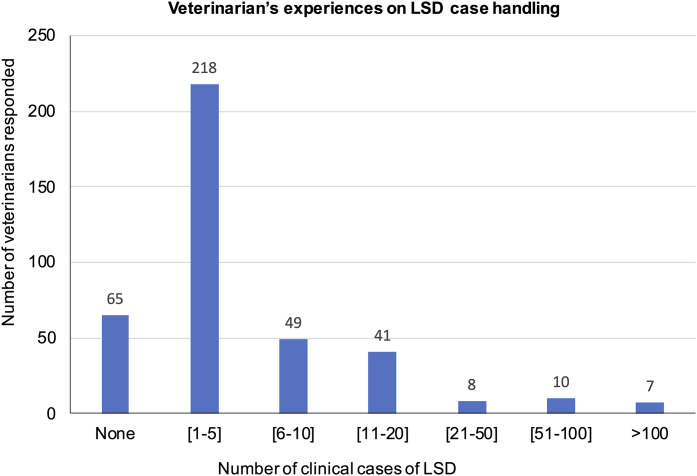
Experiences (number of cases handled) of veterinarians in managing LSD outbreaks.

Regarding experiences, particularly the number of LSD cases handled, more than half of the respondents (54.77%) reported attending to 1–5 cases (Fig 2). Smaller proportions had managed 6–10 cases (12.31%), 11–20 cases (10.30%), and over 20 cases (10.56%) in total. A few veterinarians had attended to more than 50 cases, reflecting significant clinical exposure to LSD in certain high-burden areas.

Since the participants are practicing veterinarians and lumpy skin disease in cattle is currently prevalent throughout Bangladesh, all the participants were aware of LSD and handled some clinical cases of LSD [28–29]. However, some of the participants only worked as veterinary practitioners in Dhaka city and dealt mainly with cats and dogs; that’s why about 16% of the participants had never dealt with this disease previously.

### Epidemiological risk factors of LSD

The majority of veterinarians (85.56%) acknowledged that LSD can affect cattle of all ages (Table 2), reflecting a sound understanding of the disease’s broad host range within the bovine population. This aligns with previous epidemiological findings indicating that LSD does not discriminate by age, although young calves and older animals may show varied clinical outcomes due to immune status differences. Several study findings revealed that older cattle are generally at greater risk for LSD occurrence than younger cattle [30–31]. On the contrary, a study reported that all ages are susceptible to LSD, which was consistent with veterinarians’ thoughts [32].

**Table 2:** Knowledge and attitude of veterinarians regarding epidemiological risk factors of LSD.

Risk factors	Category	Percentage % [95% CI]
Age association	All ages	85.56 [81.64 to 88.76]
	Young	9.63 [7.03 to 13.04]
	Old	4.81 [3.07 to 7.48]
Sex association	Both sexes	94.67 [91.91 to 96.52]
	Male	1.60 [0.74 to 3.45]
	Female	3.73 [2.24 to 6.17]
Breed association	No breed predisposition	67.03 [62.07 to 71.64]
	Local	12.26 [9.29 to 16.01]
	Exogenous	3.54 [2.08 to 5.97]
	Cross	13.35 [10.25 to 17.21]
	Both local and cross	3.00 [1.68 to 5.29]
	Both exogenous and cross	0.82 [0.28 to 2.38]
Presence of seasonal influences	Hot humid	75.28 [70.57 to 79.45]
	Cold season	10.83 [8.03 to 14.47]
	No influence	13.39 [10.70 to 17.38]

An overwhelming proportion (94.67%) recognized that both male and female cattle are susceptible to LSD, consistent with established knowledge that the virus affects all sexes without apparent bias. Previous studies revealed there is no significant variation between male and female animals in the occurrence of LSD [33–34]. This awareness is critical, as gender-neutral susceptibility implies that control measures and surveillance should equally target all cattle populations.

Regarding breed predisposition, most participants (67.03%) correctly indicated no specific breed susceptibility. However, a notable proportion attributed susceptibility to particular breeds, local (12.26%), crossbreeds (13.35%), and exogenous breeds (3.54%). This variation in perception may reflect regional differences in breed performance, management practices, or anecdotal observations rather than clear genetic predisposition [28,33]. Although some studies suggest that breed-related factors may influence clinical severity or recovery [32], the current consensus is that LSD affects all cattle breeds, and breed-specific susceptibility remains inconclusive.

Seasonality appeared to be well-recognized by veterinarians, with 75.28% reporting a higher incidence of LSD during the hot-humid season. Previous studies showed that the occurrence of LSD is associated with the hot-humid season when mechanical vector population is abundant [34–35]. The increased vector population during warm and humid months facilitates viral spread, emphasizing the need for heightened preventive measures during these periods. Nonetheless, 13.39% of participants believed seasonality does not affect LSD occurrence, highlighting a potential gap in understanding or local variability in disease dynamics.

The findings presented herein are perception-based and derived from a descriptive cross-sectional survey. Interpretations regarding breed predisposition, seasonal influence, treatment variability, and diagnostic confidence reflect veterinarians’ subjective assessments rather than objectively measured or causally established associations. Results reflect a variable understanding of LSD epidemiology, so there may be some knowledge gap on epidemiological risk factors of LSD among veterinarians. Continued training and dissemination of updated epidemiological data are recommended to improve the knowledge related to breed predisposition and seasonality, thereby enhancing surveillance and intervention strategies.

### Transmission routes of LSD

Veterinarians demonstrated varied perspectives on the primary transmission routes of LSD among cattle. Approximately one-third of respondents (33.69%) identified mechanical transmission via arthropod vectors as the main route, highlighting awareness of the role of insects such as flies, mosquitoes, and the role of ticks in spreading the virus (Table 3). A substantial proportion (22.19%) considered direct contact between animals as the primary mode of transmission, reflecting recognition of the importance of close physical intra-herd interactions in disease spread.

**Table 3:** Knowledge and attitude of veterinarians about the transmission dynamics of LSDV.

Transmission Mode	Number of respondents	Percentage (%) [95% CI]
Direct contact	83	22.19 [18.28 to 26.67]
Mechanical vector	126	33.69 [29.09 to 38.62]
Both direct contact and mechanical vector	63	16.84 [13.39 to 20.97]
Direct contact, mechanical vector and suckling of infected mothers’ milk	31	8.29 [5.90 to 11.53]
Direct contact, mechanical vector and through veterinary utensils/Iatrogenic	29	7.75 [5.45 to 10.91]
Both direct contact and suckling of infected mothers’ milk	16	4.28 [2.65 to 6.84]
Direct contact, mechanical vector, suckling of infected mothers’ milk and through veterinary utensils/Iatrogenic	13	3.48 [2.04 to 5.86]
Others	13	3.48 [2.04 to 5.86]

A proportion of veterinarians recognized multiple transmission routes of LSDV, including combinations of direct contact and mechanical vectors (16.84%), infected milk (8.29), and contaminated veterinary instruments (7.75), reflecting varying levels of awareness regarding the multifactorial nature of disease transmission. Other combinations, such as direct contact with suckling of infected mothers’ milk (4.28%) and all four routes combined, direct contact, mechanical vectors, suckling, and veterinary instruments (3.48%) were also reported, reflecting diverse levels of knowledge about transmission dynamics. Previous studies reported that LSDV is transmitted mainly through arthropods, particularly blood-sucking insects [36–37]. However, this virus can also be transmitted through contaminated feed and water and direct transmission in the later stages of the disease via saliva, nasal secretions, and semen [13,38]. While most veterinarians, in line with existing literature, identified the mechanical vector as a key transmission route, there remains variability in understanding the relative importance and interplay among different transmission modes. Such knowledge is critical for designing comprehensive control strategies that target not only vector control but also management practices and biosecurity measures to prevent iatrogenic spread.

### Diagnostic practices for LSD

Of the 398 participating veterinarians, 333 (83.67%) were experienced in diagnosing and managing at least one case of LSD based on clinical history and clinical signs. Among them, approximately 92% recognized the disease by observing characteristic spherical, well-circumscribed skin nodules, either alone or in combination with other lesions (Table 4). Veterinarians with diagnostic experience identified some other key clinical signs, including skin eruptions (53%), fever (52%), discharge from ruptured nodules (32%), and localized edema (24%). These symptoms were observed individually or as part of a broader clinical picture. Additional signs such as edema in the inguinal region, swelling of the limbs and joints, and respiratory distress were also reported as important diagnostic indicators. While the findings summarize the most frequently observed clinical features, it is acknowledged that LSD may present with a broader range of manifestations in the field.

**Table 4:** LSD clinical sign severity scoring model.

Clinical Sign	Frequency Reported (%)	Severity Rank*	Suggested Score**
Nodular skin lesions (round-circumscribed nodules)	~92%	Very High	3
Skin eruptions/ papules	53%	High	2.5
Fever	52%	High	2.5
Nodule rupture with discharge	32%	Moderate	2
Edema (general/localized)	24%	Moderate	2
Inguinal region swelling	<20% (estimated)	Mild-Moderate	1.5
Pain and swelling in legs/joints	<20% (estimated)	Mild-Moderate	1.5
Respiratory distress	<10% (estimated)	Mild	1

*Severity Rank: Based on visibility, impact on animal health, and diagnostic utility.

**Score: Suggested numerical value for semi-quantitative diagnosis or risk assessment.

The cumulative score can be calculated per case, with a proposed cut-off score of ≥6 indicating a “highly probable” LSD diagnosis, particularly during outbreaks.

To support systematic clinical evaluation, a hypothetical clinical sign severity ranking and scoring model for bovine LSD, developed from veterinarians’ observations, is presented in Table 4. Here, each clinical sign is assigned a severity rank and a corresponding numerical score based on its frequency, visibility, impact on animal health, and diagnostic importance. The cumulative score aims to support semi-quantitative clinical diagnosis, with a proposed threshold of ≥6 indicating a highly probable LSD case, especially in outbreak situations.

The development of a clinical sign severity ranking and scoring model represents an important step toward standardizing the clinical diagnosis of LSD in the field. Given the variability in disease presentation and the reliance on clinical signs for diagnosis, such a model can support veterinarians in objectively assessing case severity and prioritizing management strategies. Our findings indicate that certain clinical signs, particularly the presence of characteristic skin nodules and fever, are highly consistent indicators of LSD. A report by FAO also prioritized small, round, circumscribed nodules on the body as the main lesion for LSD [13]. A significant proportion of veterinarians also looked for skin eruption, fever, oozing from the eroded nodules, and edema in the body region to diagnose LSD, which are consistent with existing literature [36]. The inclusion of additional signs such as discharge from ruptured nodules and localized edema reflects the complexity of the disease’s clinical picture and its progression. By assigning weighted scores to individual clinical signs, the model enables a more objective assessment of disease severity, thereby supporting clinical decision-making, standardizing case evaluation, and enhancing communication among veterinary professionals, with potential applications in artificial intelligence (AI)-assisted veterinary practices.

However, the proposed scoring model should currently be considered only as a preliminary observational framework rather than a clinically validated diagnostic tool. Future studies should aim to refine the scoring system, incorporate additional clinical and laboratory parameters, and evaluate its predictive value in diverse epidemiological settings. Implementing such standardized tools alongside training and improved diagnostic support may ultimately contribute to better disease control, improved animal welfare, and reduced economic losses associated with LSD.

### Accuracy of the clinical diagnosis of LSD

**Fig 3:**
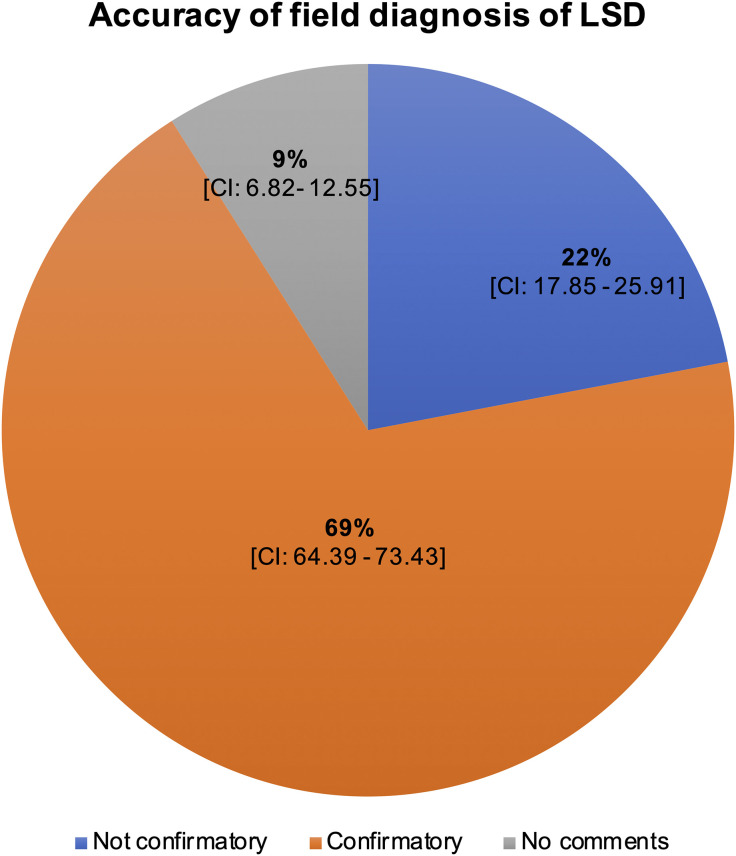
Opinion of veterinarians regarding the accuracy of field diagnosis of LSD. [CI: Confidence Interval at 95%].

Veterinary practitioners primarily diagnose LSD based on clinical signs; however, the variability and severity of these signs raise concerns regarding the accuracy of field-based diagnosis. In response to whether clinical sign-based diagnosis is sufficient, 69% of veterinarians felt this was enough to make the diagnosis and initiate treatment. Conversely, 22% of respondents indicated that additional laboratory confirmation is necessary. The remaining 9% were undecided or chose not to comment on the matter (Fig 3).

LSD diagnosis through clinical signs is a common practice among field veterinarians due to its cost-effectiveness and rapid decision-making benefits. The finding revealed that 69% of veterinarians consider clinical signs sufficient for confirmation, which indicates a high level of confidence in field diagnosis. Studies to confirm the presumptive diagnosis of LSD by molecular technique showed that about 90.14% of presumptively diagnosed samples were positive for LSD in the PCR test in Iraq [39] and 100% of the presumptively LSD diagnosed skin biopsy samples were positive in the PCR test in Egypt [40]. Therefore, the attitude of veterinarians towards relying heavily on field diagnosis to confirm LSD is somewhat upsetting.

On the other hand, the 22% of veterinarians who emphasized the need for laboratory confirmation highlight a critical understanding of the limitations of clinical diagnosis alone. The clinical manifestations of LSD can be variable and often overlap with other dermatological conditions, making differential diagnosis challenging in the field [4]. Inaccurate clinical diagnosis may lead to delayed control measures and inappropriate antimicrobial usage. Laboratory-based diagnostic methods such as PCR, virus isolation, and serological assays significantly enhance diagnostic accuracy and are essential for confirmatory diagnosis and epidemiological surveillance [41]. Recent molecular studies have further emphasized the importance of real-time PCR not only for confirmatory diagnosis but also for evaluating vaccine-induced protection and post-vaccination viral dynamics [42]. Therefore, the adoption of such confirmatory diagnostic practices will improve case identification, thereby strengthening national and regional control programs.

### Differential diagnosis of LSD

Typical acute LSD cases are highly characteristic, but early stages of infection and mild cases may be difficult to distinguish. Regarding veterinarians’ attitudes toward the differential diagnosis of LSD, only 8% of participants reported diagnosing LSD without confusion with other diseases. A majority (73.62%) acknowledged encountering diagnostic confusion during field cases. The diseases most frequently mistaken for LSD included cowpox (29.90%), papillomatosis (21.61%), and allergic skin conditions (12.81%). Additionally, 11.06% of respondents identified other conditions, such as dermatophilosis, tick bites, abscesses, tumors, hemorrhagic septicemia, and bovine ephemeral fever, as potential sources of diagnostic confusion. Notably, 18.34% of participants were undecided or refrained from commenting on this issue. The variability in clinical presentation often leads to diagnostic challenges, as other diseases may exhibit similar clinical manifestations [43]. A report by FAO mentioned insect bites, urticaria, photosensitization, pseudo cowpox, dermatophilosis, and bovine herpes mammillitis as a differential diagnosis for LSD [13]. Findings of the present study underscore the substantial challenges veterinarians face in distinguishing LSD from clinically similar diseases, which may hinder timely and accurate diagnosis and, consequently, effective disease management. High reliance on clinical diagnosis despite confusion may lead to empirical and prophylactic antibiotic administration, which is considered one of the root causes of poor antimicrobial stewardship. Therefore, confirmatory PCR diagnostics are essential, not just for surveillance, but to provide veterinarians with the confidence to avoid unnecessary antibiotic use in ambiguous clinical cases, thereby directly fighting off antimicrobial resistance.

### Therapeutic practices for LSD

Participating veterinarians reported a diverse array of treatment protocols for controlling LSD, highlighting considerable variability in clinical practices (Table 5). The most frequently employed regimen, reported by 13.32% of respondents, consisted of a combination of antibiotics, antihistamines, and non-steroidal anti-inflammatory drugs (NSAIDs). Other frequently reported treatment combinations included antibiotics, antihistamines, NSAIDs, and antiseptic dressings (6.03%), as well as antibiotics, antihistamines, NSAIDs, and vitamin C supplementation (4.02%).

**Table 5:** Treatment practices followed by veterinarians for LSD cases.

Treatment Protocol/ Drug Used	Number of Respondents	Percentage (%) [95% CI]
Antibiotics + Antihistamines + NSAID	53	13.32 [10.14 to 17.05]
Antibiotics + Antihistamines + NSAID + Antiseptic dressing	24	6.03 [3.90 to 8.84]
Antibiotics + Antihistamines + NSAID + Vitamin C	16	4.02 [2.31 to 6.45]
Antibiotics (in any combination)	239	60.05 [55.05 to 64.90]
Antihistamines (in any combination)	230	57.79 [52.77 to 62.69]
Anti-inflammatory/NSAID (in any combination)	202	50.75 [45.73 to 55.77]
Antiseptic dressing	103	25.88 [21.64 to 30.48]
Antiviral drug	96	24.12 [20.00 to 28.64]
Vitamin C	86	21.61 [17.66 to 25.98]
Zinc preparation	57	14.32 [11.03 to 18.15]
Neem extract + Molasses + Sodium bicarbonate	37	9.30 [6.63 to 12.59]
No comment on treatment	65	16.33 [12.84 to 20.34]

Overall, a majority of veterinarians (60.05%) indicated prescribing antibiotics alongside other therapeutic agents for LSD cases. Antihistamines and anti-inflammatory drugs were also widely used, with 57.79% and 50.75% of respondents reporting their administration, respectively. Additional treatments included antiseptic dressings (25.88%), antiviral drugs (24.12%), vitamin C (21.61%), and zinc supplements (14.32%). Notably, 9.30% of veterinarians reported employing traditional remedies, such as a mixture of neem extract, molasses, and sodium bicarbonate. A substantial minority (16.33%) did not disclose any information regarding their treatment approaches for LSD (Table 5).

Because of viral etiology, there is no specific treatment for this contagious disease [44]. Symptomatic treatment is the only tool for temporary relief, like anti-inflammatory drugs, which act as analgesics and appetite inducers, and antihistaminic drugs tend to reduce the allergic reaction [45]. Treatment approaches may differ from one doctor to another according to the clinical severity of the disease, duration of illness, and economic considerations of the owners. While antibiotics were the most commonly prescribed medication, often in combination with antihistamines and non-steroidal anti-inflammatory drugs (NSAIDs), their widespread use in a viral disease raises concerns about the rationality of antimicrobial administration [45]. Although antibiotics may be justified in cases with secondary bacterial infections, indiscriminate use can contribute to antimicrobial resistance (AMR), a growing global health threat. The frequent use of antihistamines and NSAIDs is consistent with attempts to manage the inflammatory and allergic symptoms associated with LSD. The inclusion of supportive therapies such as vitamin C, zinc supplements, and antiseptic dressings indicates efforts to promote recovery and prevent secondary infections [45–46]. Additionally, topical antiseptics may be applied to the patient as a skin preparation to reduce the risk of skin infection [47]. Notably, a minority of veterinarians reported using traditional remedies, highlighting the role of ethnoveterinary practices in certain regions. The findings reveal considerable variability in the treatment protocols employed by veterinarians for managing LSD, reflecting a lack of standardized clinical guidelines. Furthermore, the prevalent use of antibiotics raises concerns about rational antimicrobial use, especially given the primarily viral etiology of LSD and the risk of antimicrobial resistance development. These findings underscore the need for evidence-based treatment guidelines and targeted training programs to harmonize veterinary practices and promote prudent use of therapeutics in LSD management. In addition, vaccination remains the most effective strategy for controlling LSD; however, vaccine choice and efficacy may vary. Experimental evidence indicates that homologous vaccines based on lumpy skin disease virus (LSDV) provide superior protection compared to heterologous vaccines such as sheep pox virus. For instance, Shafik et al. [48] demonstrated that LSDV vaccines conferred better clinical protection and reduced disease severity against field isolates compared to sheep pox–based vaccines, and highlighted the importance of selecting appropriate vaccine strains for effective disease control.

### Post-treatment complications (sequelae) of LSD cases

**Fig 4:**
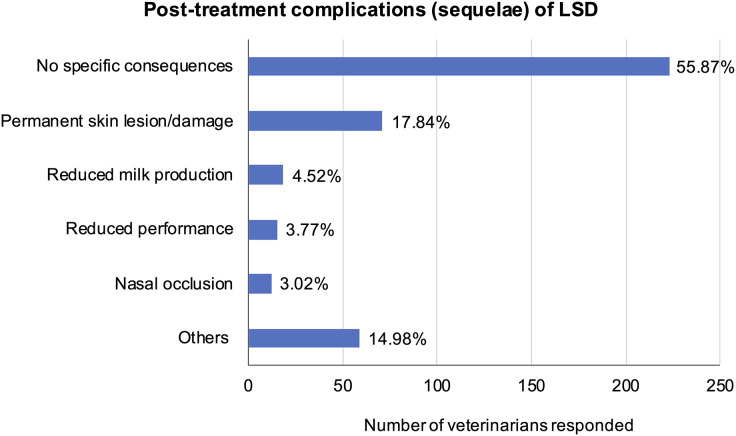
Post-treatment complications (sequelae) of LSD cases.

LSD significantly impacts cattle health, causing considerable morbidity during the acute phase and frequently leading to chronic conditions even after successful treatment. In response to attitude and knowledge on morbidities and complications associated with LSD, about 55.78% of the participants replied that they didn’t experience any specific consequence caused by LSD. Some participants observed consequences like skin damage (17.84%), reduced milk production (4.52%), reduced overall performance (3.77%), nasal occlusion (3.02%), and other sequels (14.98%), including abscess, lameness, maggot, weight loss, and reduced fertility, either separately or in combination (Fig 4).

More than half of the participants (55.78%) reported no direct experience with post-infection sequelae, which may reflect regional variations in disease course and duration or differences in veterinary diagnostic and reporting practices. Commonly reported sequelae such as skin damage, secondary bacterial infection leading to abscess and myiasis, reduced milk production, and decreased overall performance are consistent with existing literature describing the economic and welfare consequences of LSD [35]. Moreover, less frequently observed outcomes, such as abscess formation, lameness, maggot infestations, weight loss, and reduced fertility, underscore the complex and multifactorial nature of the disease’s long-term impact on affected cattle. Improving veterinarians’ capacity to identify and manage these sequelae is vital for reducing the prolonged burden of LSD, promoting animal welfare, and minimizing economic losses. Additionally, this knowledge is crucial for informing livestock owners about potential complications and encouraging early, appropriate intervention strategies.

### Therapeutic practices of antibiotics for LSD and the risk of antimicrobial resistance

We checked out the perception and attitude of veterinarians towards the rational use of antimicrobials for LSD treatment in the field and their possible consequences in the development of antimicrobial resistance. Among the surveyed veterinarians, only 43.22% believed that antibiotics are used rationally for the treatment of LSD (Table 6). In contrast, nearly one-third (32.16%) perceived that antibiotics are not used rationally in LSD management, while 24.62% of participants reported having no opinion or knowledge regarding the rational use of antibiotics for this disease.

**Table 6:** Perception and attitude of veterinarians towards the antibiotic therapy for LSD management and their consequences for developing antimicrobial resistance.

Query	Responses	Percentage (%) [95% CI]
Are antibiotics used rationality in for treating LSD?	Yes	43.22 [38.44 to 48.12]
	No	32.16 [27.76 to 36.90]
	No idea	24.62 [20.65 to 29.08]
Is there any possibility of developing antimicrobial resistance due to antibiotics usage in LSD treatment?	Yes	62.06 [57.20 to 66.69]
	No	22.86 [19.01 to 27.24]
	No idea	15.08 [11.89 to 18.92]

The majority of respondents (62.06%) acknowledged that the use of antimicrobials in treating LSD cases contributes to the development of antimicrobial resistance (AMR). However, 22.86% of veterinarians did not consider antimicrobial use in LSD treatment to be a factor in AMR development, and 15.08% were uncertain about this relationship. These findings reveal a significant gap in awareness and consensus regarding the appropriate use of antibiotics in LSD management and the potential implications for AMR [49]. Rational antimicrobial use is critical to minimizing the emergence of resistant pathogens, particularly in veterinary settings where indiscriminate or prophylactic antibiotic use is common. The high proportion of veterinarians acknowledging the risk of AMR suggests increasing awareness but also underscores the need for continued education and stewardship programs to promote prudent antimicrobial use in LSD and other infectious diseases in livestock [49,50]. It should be noted, however, that the present study did not capture data on specific antibiotics prescribed, which substantially limits the depth of stewardship-related conclusions that can be drawn and warrants investigation in future studies.

The survey findings highlight critical areas for intervention to promote rational antimicrobial use in LSD management and mitigate AMR risks. Given that less than half of the veterinarians believe antibiotics are used rationally for LSD, and a substantial proportion remain uncertain about the link between antimicrobial use and resistance development, targeted educational programs are urgently needed [49]. From a policy perspective, collaboration among veterinary authorities, public health agencies, and livestock producers is essential to foster responsible antimicrobial use and slow the emergence of resistance [49,50]. Regulatory frameworks should incentivize prudent antibiotic use and restrict over-the-counter sales without prescription. Moreover, integrating AMR awareness into routine veterinary education and continuing professional development will ensure sustained impact. Addressing these issues in the context of LSD management will contribute to safeguarding antimicrobial efficacy, improving animal health outcomes, and protecting public health.

### Limitations

This study has several limitations that should be considered when interpreting the findings. First, data were self-reported, which may introduce recall and social desirability bias. Second, the sample was skewed toward veterinary fresh graduates and early-career veterinarians, with potential under-representation of private practitioners, limiting the statistical power of exploratory comparative analyses performed and the generalizability of subgroup comparison. Future studies incorporating greater demographic diversity and pre-specified inferential frameworks would generate more robust evidence for targeted policy interventions. Third, clinical LSD diagnoses were not confirmed by PCR, restricting the assessment of diagnostic accuracy. Fourth, although 60.5% of respondents reported using antibiotics, the specific drugs or classes were not captured, limiting the evaluation of antimicrobial stewardship. Finally, sampling constraints associated with voluntary participation may affect representativeness. Collectively, these limitations highlight the need for future studies incorporating laboratory-confirmed cases into the proposed clinical sign scoring model, a more balanced representation of veterinarians across experience categories and practice sectors, and detailed antimicrobial usage data, including specific drugs, dosage, and route of administration, to enable a more rigorous evaluation of prescribing practices and their implications for antimicrobial resistance in food animal settings.

## Conclusion

Bangladeshi field veterinarians exhibited a shared awareness of LSD, with some demonstrating a thorough understanding, while others displayed gaps in knowledge, particularly regarding risk factors and transmission dynamics. Their attitudes reflected a spectrum of perceptions and beliefs, indicating varying degrees of recognition of the disease’s severity and its impact on cattle health and productivity. The study also revealed unstructured and inconsistent treatment approaches among practitioners, emphasizing the need for diagnostic standardization, laboratory support, stewardship-guided standardized treatment protocols for LSD, AMR stewardship training, and capacity-building initiatives. Strengthening veterinary competencies through standardized education and guidelines could greatly improve the uniformity and effectiveness of LSD management across the country.
